# Game Theoretic Approach for Systematic Feature Selection; Application in False Alarm Detection in Intensive Care Units

**DOI:** 10.3390/e20030190

**Published:** 2018-03-12

**Authors:** Fatemeh Afghah, Abolfazl Razi, Reza Soroushmehr, Hamid Ghanbari, Kayvan Najarian

**Affiliations:** 1 School of Informatics, Computing and Cyber Systems, Northern Arizona University, Flagstaff, AZ 86011, USA; 2 Department of Emergency Medicine, University of Michigan, Ann Arbor, MI 48109, USA

**Keywords:** false alarm reduction, intensive care units, feature selection, coalition game theory, Banzhaf power

## Abstract

Intensive Care Units (ICUs) are equipped with many sophisticated sensors and monitoring devices to provide the highest quality of care for critically ill patients. However, these devices might generate false alarms that reduce standard of care and result in desensitization of caregivers to alarms. Therefore, reducing the number of false alarms is of great importance. Many approaches such as signal processing and machine learning, and designing more accurate sensors have been developed for this purpose. However, the significant intrinsic correlation among the extracted features from different sensors has been mostly overlooked. A majority of current data mining techniques fail to capture such correlation among the collected signals from different sensors that limits their alarm recognition capabilities. Here, we propose a novel information-theoretic predictive modeling technique based on the idea of coalition game theory to enhance the accuracy of false alarm detection in ICUs by accounting for the synergistic power of signal attributes in the feature selection stage. This approach brings together techniques from information theory and game theory to account for inter-features mutual information in determining the most correlated predictors with respect to false alarm by calculating Banzhaf power of each feature. The numerical results show that the proposed method can enhance classification accuracy and improve the area under the ROC (receiver operating characteristic) curve compared to other feature selection techniques, when integrated in classifiers such as Bayes-Net that consider inter-features dependencies.

## 1. Introduction

As there is no single sensor/device capable of complying with all clinical requirements, multiple therapeutic and monitoring devices are often deployed in the Intensive Care Units (ICUs) to collect real-time data for diagnosis, prognosis, treatment and more generally, patient monitoring. These devices generate visual and acoustic alarms to inform nurses and physicians about changes in a patient’s condition or a failure in device functionality [1]. However, the rate of false alarm generation is too high, which can result in disrupting the monitoring procedure in severe situations, alarm fatigue and desensitization of clinical staff to the alarms and hence cause ignorance or delay in reaction to true alarms [2,3]. As reported in [4,5], caregivers are usually overwhelmed with 350 alarm conditions per patient per day, of which 80–99% are meaningless or false [6,7,8]. Therefore, false alarms are considered the top hazard imposed by the use of medical technologies [9,10,11].

A false alarm might happen due to the low quality of signals [2] as a result of several factors such as noise, motion artifacts, missing data, and technical defects. Various methods have been proposed to reduce false alarms [1,12,13,14,15,16,17,18], which can be generally classified into learning and non–learning methods. In the learning category, a labeled dataset is usually available and a set of features is extracted from the dataset to train a model using a portion of the dataset. Then, this model is tested and validated using a validation technique. Imhoff et al. [1] have reviewed a number of learning and statistical approaches and discussed their potential use for clinical applications, particularly, false alarm reduction. Behar et al. [17] have designed a support vector machine (SVM)-based method to estimate the quality of an electrocardiogram (ECG) segment using signal quality indecies (SQIs). SQIs are used to assess the quality of a signal or its level of noise. This model could reduce the number of false alarms as it can eliminate low quality ECG segments with high accuracy. Gambarotta et al. [2] have reviewed the techniques on quality scoring of ECG and arterial blood pressure (ABP) signals and also surveyed the algorithms that exploits the relationship among ECG, ABP and photoplethysmogram (PPG) to reduce the false alarm rate. Among the learning methods proposed in the literature, they referred to SVM, multilayer perceptron (MLP), naive Bayes and linear discriminant analysis (LDA). Ansari et al. [16] performed band-pass filtering on ECG and pulsatile signals and also trend estimation on ECG signals. They applied different QRS-complex detection methods and classified beats using a decision tree approach. Finally they developed another decision tree classifier to classify true and false alarms. Antink et al. [13] applied band-pass filtering, peak detection, fast Fourier transform (FFT), principle component analysis (PCA), and some statistical analyses and extracted a number of features to train machine learning methods. They applied four classifiers: random forest, SVM binary classification decision tree and regularized linear discriminant analysis for classifying alarms. Zhang and Szolovits used ECG, plethysmography, blood pressure, venous and arterial oxygen saturation and oxygen perfusion as features and trained a classification tree and also artificial neural networks to classify the alarms. They showed that training with eight hours of the data can result in better performance compared with standard thresholding methods [19]. Li and Clifford [18] extracted 147 features and SQI metrics from ABP, ECG, Spo2 and PPG and trained a random forest classifier. They used the 10-fold cross validation technique and achieved the sensitivity of 100% and specificity of 24.5%. Salas-Boni et al. [20] used ECG signal and applied wavelet transform to extract the features. They developed a logistic regression classifier using L1-regularized and achieved a false alarm suppression of 25.5% without suppressing true alarms.

Among the non–learning methods, we can refer to the method proposed in [21]. In this method, a wavelet transform is applied to the ECG signal to remove its noise. Then, the quality of vital signals (ECG and ABP) in intensive care patients is measured using SQIs. After that the combination of SQI, Heart Rate Variability (HRV) and ABP is used for the judgment of false alarms. Delayed activation of alarms is another simple approach to decrease the false alarms [6,22,23]. Scmid et al. [22] and Teo et al. [24] used ECG, ABP and PLETH signals and designed a majority voting approach with a fixed threshold to determine if an alarm is true or not. Aboukhalil et al. [15] used a database of MIMIC II to analyze five types of ECG arrhythmia. They developed an algorithm based on the morphological and timing information of ABP signal and achieved the false alarm suppression rate of up to 42.7%. Li and Clifford [25] proposed an SQI to assess the quality of ABP signal and reject the noisy ones. They estimated the ABP-derived HR and compared it with the monitor’s HR threshold and rejected the false HR-related arrhythmia. They could reduce the false alarm rates of extreme bradycardia and extreme tachycardia to 74.13% and 53.81%, respectively.

One of the challenges facing the above mentioned methods is that features whose impact on the model performance is individually low might be excluded in the feature extraction phase, while their combination with other features could improve the overall performance. These methods consider either the effect of each feature by itself on the target or the inter-feature mutual information to improve the performance. Therefore, the features relevant to the target class might be discarded if they have high correlation to the already selected features.

To suppress the false alarm in ICUs, here we develop a new coalition game-theoretical model based on *Banzhaf power* index that accounts for interdependency among the extracted features and their relevancy to the target class. Coalition game theory has been recently employed in the feature selection stage of machine learning approaches to improve their performance, where features are modeled as game players [26,27,28,29,30,31]. In the majority of these existing game-theoretical approaches, the importance of features on classification accuracy is measured by *Shapley value*. The Shapley value of a feature shows the contribution of the feature in improving the accuracy of classification when all possible coalitions of features with any arbitrary size are considered. While this method can have a considerable impact on capturing the higher-level correlation among features (e.g., more than mutual correlation between two features), it involves a high computational complexity to calculate this factor for all possible groupings of the features, in particular in the presence of a large number of features. In [27], we utilized a game theoretic feature selection method based on Shapley value to select a combination of features that enhance the hemorrhage severity prediction over a heterogeneous data set to predict. We considered all possible coalitions of size 4–10 due to intractable computational complexity of calculating Shapley value over larger coalitions and computed the importance of each feature using multi-perturbation Shapley value.

In [29], we studied the problem of false alarm reduction in ICUs, where three main signals; electrocardiogram (ECG), plethysmogram (PLETH), arterial blood pressure (ABP), were used to classify alarms to false and true. In the first stage, we calculated wavelet coefficients at different levels of decomposition for each of the mentioned signals. Then, we extracted a number of statistical and information theory-based features from the coefficients of wavelets at each level. A Shapley value-based feature selection approach was utilized to reduce the possibility of removing high-impact features that are highly correlated with other selected ones. While the Shapley value was only calculated for small size coalitions, the feature selection method still involved a considerable computational complexity. More importantly, considering smaller coalitions of features resulted in reducing the accuracy of the alarm detection model. To address these challenges, in this paper, we propose a new game-theoretic feature selection method based on utilizing *Banzhaf power* to declare salient features with comparable accuracy but much less complexity. This metric is proportional to the number of times that a feature is a critical player for a coalition. In the proposed model, we define an information-theoretic notion for Banzhaf power, where a feature is determined to have a critical impact on a set of features if it increases the relevancy of the selected feature set on a target class and also is interdependent on more than half of the members in the set. The numerical results validate the desirable performance improvement of this method in reducing the false alarm rate compared to existing feature selection techniques when a classification method that has the capability of considering inter-features dependencies is utilized.

The rest of this paper is organized as follows. List of the abbreviations used in this paper is presented in Section 5. In Section 2, an introduction to the data set studied in this work is provided. Section 3 describes the proposed feature extraction techniques and signal analysis. The proposed coalition-based game theoretic feature selection method based on Banzhaf power is presented in Section 4. In Section 5 we present the results of numerical analysis and finally conclusion remarks are given in Section 6.

## 2. Description of Data Source

In this work, we use the publicly available Physionet Challenge 2015 database [32,33]. Four hospitals in the USA and Europe have been involved in producing the database. The definition of the alarms is presented in Table 1 [32]. Measurement for three vital signals of ECG-II, APB, and plethysmogram (PLETH) are utilized where each alarm is labeled as *true*, or *false*. Each alarm was reviewed by a team of experts and at least two of them agreed on the alarm type. These alarms are assumed to be at least 5 min apart and are triggered 5 min from the start of each record while the onset of the events is within 10 s of the alarm (i.e., between 4:50 and 5:00 of the record). The PhysioNet challenge-2015 dataset includes a training dataset containing the recordings for 750 patients, and a test dataset containing the recordings for 500 patients. It is worth mentioning that the test dataset is not publicly available and we only had access to the training dataset. Out of 750 recordings in training dataset, only 220 samples include all three signals of ECGII, plethysmogram (PLET) and ABP that were used in this study. The resolution and frequency of each signal are 12 bit and 250 Hz, respectively. Furthermore, each signal has been filtered by notch filters and a finite impulse response (FIR) band pass (0.05 to 40 Hz). The signals might suffer from movement artifact, sensor disconnects, interference from pacemakers and other events.

## 3. Signal Analysis and Feature Extraction

Extracting relevant features from the entire time-series signals is a key step in detecting the false alarms, as considering the original signals results in a large number of highly correlated features compared to the sample size that increases the chance of over–fitting the model to the training data. Here, we apply discrete wavelet transforms (DWT) on ECG, ABP and PLETH signals. This method is utilized as it can separate details in signals compared to other transforms and it can eliminate the noise with a low distortion rate. The DWT’s capability to detect specific time-frequency components of ECG signals has motivated several researchers to utilize this method in several related applications [34,35,36].

This transform performs an adaptive time-frequency decomposition of patterns in a signal. Moreover, the signal can be represented by a few wavelet coefficients and hence less features can be extracted from a signal.

A set of dilated-translated wavelets, $\psi_{a,b}$, can be defined as: [37]. DWT components are shifted and scaled versions of the mother wavelet defined as:

(1) $$\psi_{a,b}\left(t\right)=\frac{1}{\sqrt{a}}\psi \left(\frac{t-b}{a}\right)$$

where a,b are scale/dilation and shift/translation parameters, respectively. There are a number of wavelet functions with different characteristics such as symmetry, vanishing moment and so on that can be used as the ψ function. Here we choose Daubechies wavelets class D−2N for analyzing the signals defined as:

(2) $$\psi \left(t\right)=\sqrt{2}\sum_{k}{\left(-1\right)}^{k}h_{2N-1-k}\times \phi \left(2t-1\right),$$

$$\phi \left(t\right)=\sqrt{2}\sum_{k}h_{k}\times \phi \left(2t-k\right)$$

In Equation (2), *h* is a high pass filter. When this filter is convolved with a signal at low scales, the output is called an approximation set of the signal. Convolving a low–pass filter, $g_{k}=h_{2N-1-k}$, at high scales generates another set called detail coefficients. Decomposing a signal to *approximate* and *detail* coefficients can be done again depending on how much detail is required. Approximate and detail coefficients can be obtained respectively from Equations (3) and (4)

(3) $$a_{i}\left(t\right)=\sum_{k}a_{i-1}\left(t\right)h_{2t-k}$$

(4) $$d_{i}\left(t\right)=\sum_{k}a_{i-1}\left(t\right)g_{2t-k}$$

In Equations (3) and (4), $a_{-1}$ shows an input signal (i.e., ABP, ECG, or PLETH). We show the calculated coefficients as $X=[E_{1},\ldots ,E_{l},A_{1},\ldots ,A_{l},P_{1},\ldots ,P_{l}]$, where *l* shows the decomposition level. $E_{i}$, $A_{i}$ and $P_{i}$, show both detail and approximate wavelet coefficients for ECG, ABP and PLETH signals respectively. For the detail coefficients i=l, and for the approximate coefficients i≠l.

Here we calculate wavelet coefficients at 6 levels (i.e., l=6) and use Daubechies-8 (db8) for analyzing ECG signals and Daubechies 4 for analyzing ABP and PLETH signals. The reason that we choose these wavelets is because of having a good match between the shape of those signals and the corresponding wavelets.

The entire 5-min recordings of these signals are used to calculate the DWT, since higher-order wavelet transforms using short signal duration does not provide informative features.

Here, we extract information–theoretic and statistical features from the wavelet coefficients as mentioned in Table 2 instead of using all the coefficients that might result in over-fitting. For computing information-theoretic properties such as entropy, we first discretized the coefficients using quantization levels obtained from Lloyd’s algorithm [38] and then used the empirical distribution as an estimate for the unknown probability distribution from which the coefficients are derived.

The first 10 features in Table 2, are typical statistical properties of the signal. Also, $\mu_{3}$ and $\mu_{4}$ are respectively the 3th and 4th standardized sample moment calculated as:

(5) $$\begin{array}{c} \mu_{n}=\frac{\Sigma_{i=1}^{N}{\left(X_{i}-\bar{X}\right)}^{n}}{N} \end{array}$$

where $\bar{X}=\frac{\Sigma_{i=1}^{N}X_{i}}{N}$, and $X_{1},\cdots ,X_{N}$ are the *N*th coefficients associated with each signal. *Kurtosis*, defined as $\kappa \left(X\right)=\frac{\mu_{4}\left(X\right)}{\sigma_{4}\left(X\right)}$, is the standardized fourth population moment about the mean measuring the peakedness of distribution. *Skewness*, defined as $\lambda \left(X\right)=\frac{\mu^{3}\left(X\right)}{\sigma^{3}\left(X\right)}$, shows how symmetric a distribution is around zero. Furthermore, *Harmonic mean* or *H mean* is defined as $\frac{N}{\sum_{i=1}^{N}1/X_{i}}$. *Interquartile range* is computed based on the difference between the 25th and 75th percentiles. *Shannon entropy of energy*, calculated as $H\left(X^{2}\right)=-\sum_{i=1}^{N}X_{i}^{2}\log_{2}\;X_{i}^{2}$, shows the entropy of the energy of the coefficients and *Log energy* is defined as $\sum_{i}\log X_{i}^{2}$. Finally, $n_{T(\alpha )}$ shows the number of wavelet coefficients larger than α.

(6) $$\begin{array}{c} n_{T}\left(\alpha \right)=\sum_{i=1}^{N}1(|X_{i}\left|>\alpha \right) \end{array}$$

where, 1(.) shows the indicator function. These features collectively capture the properties of the signal at different decomposition levels and are used as input for the proposed feature selection method.

## 4. Proposed Coalition Game-Theoretic Feature Selection Method

In this section, we first briefly describe the coalition game theory and then present the proposed feature selection method using Banzhaf power. *Coalition game* or *cooperative game* refers to a class of game theoretical approaches that study the set of joint actions taken by a group of players. This is different from non-cooperative games in which players act individually [39,40,41]. Outcome of a coalition game is defined by how players form coalitions and how the coalition payoff is divided among its members [42].

A coalition game can be defined with a pair of (N,v), where $N=\{F_{1},F_{2},\cdots ,F_{n}\}$ is the set of players with cardinality of *n* (i.e., |N|=n). The *characteristic function*, *v*, is a function representing the total payoff gained by the members of this coalition and is defined on the set of all coalitions, $v:2^{N}\to R$. We use transferable utility coalition (TU-coalition) game for which the following conditions hold for the characteristic function, *v*.

1. v(ϕ)=0 where ϕ an empty coalition.
2. $v\left(S_{i}\cup S_{j}\right)\geq v\left(S_{i}\right)+v\left(S_{j}\right)$ where $S_{i}$ and $S_{j}$, ($S_{i},S_{j}\subseteq N$) are two disjoint coalitions.

Different solutions have been defined to measure the role (importance) of a player in a transferable utility coalition game including *Shapley value* [43], *Banzhaf power* [44], and *Banzhaf value* [45]. In our proposed feature selection method, the importance of the features is measured using *Banzhaf power*. To define this metric, we first need to introduce the concept of *simple game*.

A simple game refers to a class of coalition games with characteristic function satisfying the following conditions [46].

1. v(S)∈{0,1}, For all S⊂N,
2. v(ϕ)=0, v(N)=1, and
3. For S,T⊂N, if S⊂T, then V(S)≤v(T) (monotonicity).

Based on the first property, the coalitions are divided into two sets of winning coalition, W(v)={S⊂N| v(S)=1} and losing coalition defined as L(v)={S⊂N| v(S)=0}. In these games, a player $F_{i}$ is called a *swinger* if the removal of this player from a winning coalition S converts it to a losing coalition, meaning that v(S)=1 and $v(S\backslash \left\{F_{i}\right\})=0$.

The Banzhaf power for player $F_{i}$, $\beta_{i}\left(v\right)$ represents the fraction of times that player has a critical role in converting a losing coalition to a wining one and is defined as the expectation of player $F_{i}$ to be a swinger in a simple game model assuming that formation of all coalitions are equally probable as defined below,

(7) $$\begin{array}{c} \beta_{i}\left(v\right)=\frac{\eta_{i}\left(v\right)}{2^{n-1}} \end{array}$$

where $\eta_{i}\left(v\right)$ counts all coalitions for which the player $F_{i}$ is a swinger (i.e., $\left\{S:S\subset N\backslash \left\{F_{i}\right\},\nu \left(S⋃\left\{F_{i}\right\}\right)-\nu \left(S\right)=1\right\}$).

Next, we discuss our proposed coalition-based feature selection method, in which the features are considered as the players of the game, and the *v* function is calculated based on its members (features)’s contribution to the classifier performance. We measure the contribution of each feature in the game noting all possible coalitions of the players using Banzhaf power. The criterion to determine the most informative subset of features is the relevance of this set to the target class as well as the interdependence among the group members. If the relevance of the feature $F_{i}$ on target class *C*, $R(F_{i};C)$ is defined by their mutual information, $R\left(F_{i};C\right)=I\left(F_{i};C\right)$, the relevance of coalition S on target class *C* can be approximated as [47]:

(8) $$\begin{array}{c} R\left(S;C\right)≊\frac{1}{\left|S\right|}\sum_{F_{j}\in S}\left[I\left(F_{j};C\right)\right], \end{array}$$

Likewise, the change of relevance of a coalition S on target class *C* due to the knowledge of feature $F_{i}$, $\left(F_{i}\notin S\right)$ is approximately

(9) $$\begin{array}{c} I\left(S;C|F_{i}\right)≊\frac{1}{\left|S\right|}\sum_{F_{j}\in S}\left[I\left(F_{j};C|F_{i}\right)-I\left(F_{j};C\right)\right], \end{array}$$

Moreover, two features $F_{i}$ and $F_{j}$ are defined to be interdependent of each other if the relevance between $F_{j}$ and the target class *C* is increased when $F_{i}$ ($I\left(F_{j};C|F_{i}\right)>I\left(F_{j};C\right)$), meaning that the impact of this feature cannot be overlooked in the model [48]. Parameter $\gamma_{S}^{i}$ is defined to count the number of features in coalition S that are interdependent on feature $F_{i}$ as follows

(10) $$\begin{array}{c} \gamma_{i}^{S}=1\left(I\left(F_{j};C|F_{i}\right)>I\left(F_{j};C\right)\right),\mathrm{for}\;\mathrm{all}\;F_{j}\in S. \end{array}$$

where 1(.) is the indicator function.

In order to select the most informative subset of features, we first determine the impact of feature $F_{i}$ on all possible coalitions of features excluding $F_{i}$, $\left\{S:S\subset N,F_{i}\notin S\right\}$. Feature $F_{i}$ is a swinger for coalition S, if it increases the relevance of this coalition on the target class and also if it is interdependent with at least half of the members of coalition S. Then, a swinger index $\zeta_{i}$ for feature $F_{i}$ is defined as:

(11) $$\begin{array}{c} \zeta_{i}=\left\{\begin{array}{cc} 1, & I\left(S;C|F_{i}\right)\geq 0,\gamma_{i}^{S}\geq \frac{\left|S\right|}{2}\\ 0, & \mathrm{otherwise} \end{array}\right. \end{array}$$

Consequently, the Banzhaf power of feature $F_{i}$ calculates the ratio of all coalitions for which player $F_{i}$ is a swinger, $\eta_{i}\left(v\right)=\frac{1}{2^{n-1}}\sum_{S\subset N\backslash i}\zeta_{i}^{S}$. This parameter quantifies the power of features in turning the losing coalitions into winning ones and hence can be used to choose the top informative features.

## 5. Numerical Analysis, Discussion, and Limitations

In this section, we examine the utility of the proposed approach in selecting informative features from three signal sources to verify alarm validity. For this study, we use the Physionet Challenge 2015 database as described in Section 2. The dataset includes the recorded signals for 750 patients. Out of which, only for 220 patients all three signals of ECG II, ABP and PLET are available. Therefore, we used these 220 samples to demonstrate the capability of the proposed method in extracting the correlation among different signals. We arbitrarily used 10-fold cross-validation to train the classifier (198 training samples). In order to calculate time-frequency information at different resolutions, we first apply six-level wavelet decomposition using Daubechies 8 (db8) to signals. As there are 3 signals and six levels of wavelet decomposition in each sample, we have 18 vectors of wavelet coefficients. We extract 20 statistical features as well as information-theoretic ones from each vector, and hence we have a total of 360 features. The list of features are provided in Table 2.

The proposed coalition game based on Banzhaf power evaluates the average marginal importance of each feature when joining any potential coalition of features. The metric we use is the interdependency of newly added features with the coalition members as defined in Section 4. In order to obtain interdependency, we first discretized the wavelet coefficients. The quantization levels are obtained from Lloyd algorithm, which minimizes the MSE error between the continuous values and the quantized versions for a training dataset and a given number of quantization levels (here we choose five quantization levels) [38]. The quantized values are used to calculate the required mutual information which further is used to calculate features’ interdependencies. Then, a swinger index $\zeta_{i}^{S}$ for each feature $F_{i}$ with respect to coalition S is set to 1 if the feature is interdependent with at least half of the coalition members Equation (11). The Banzhaf power for each feature $F_{i}$ is calculated as the ratio of coalitions for which the feature $F_{i}$ is a swinger. We rank the features based on their Banzhaf powers and choose the top-20 features.

**Figure 1:**
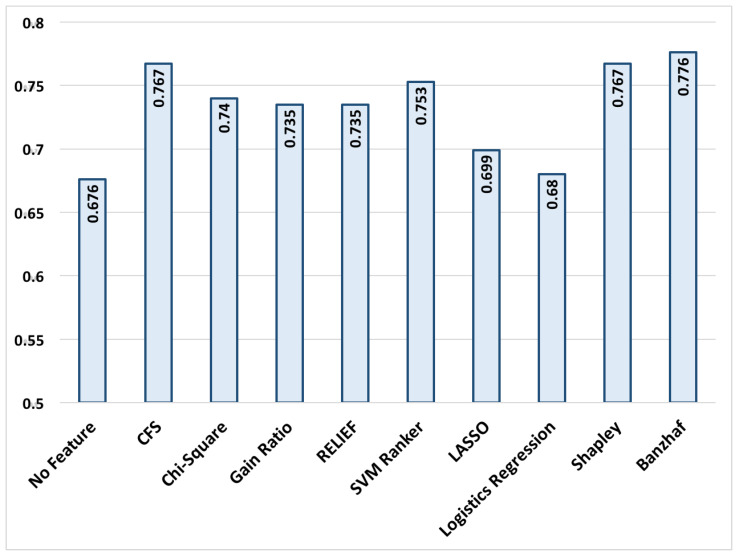
Alarm classification success rate for various feature selection methods using top-20 features. Bayes-Net classification with 10-fold cross validation is used to classify alarms into true and false alarms.

**Figure 2:**
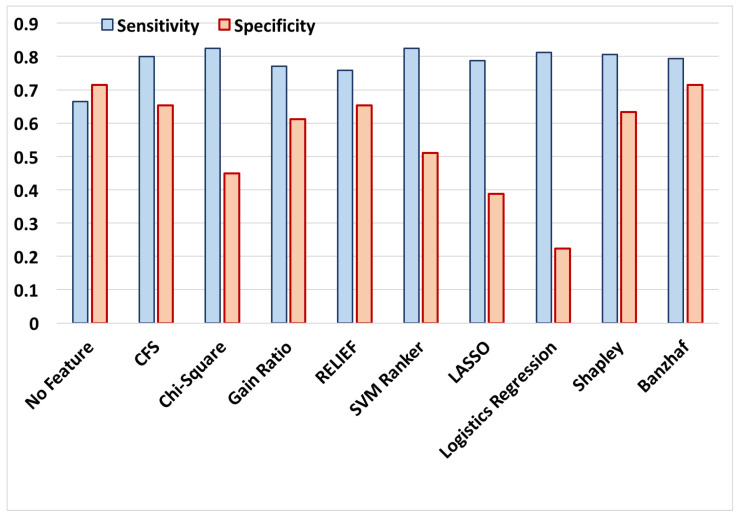
Sensitivity and specificity of various feature selection methods using top-20 features and Bayes-Net classification with 10-fold cross validation.

In order to evaluate the relevance of the obtained features, we used *Weka ver. 3.6* package and applied state-of-the-art feature selection methods to the extracted features and selected the top-20 of them for each method. In this experiment, the following attribute selection techniques are utilized: (i) A subset of features with the lowest intra correlation and the highest inter correlation with the labels are selected using the Correlation-based Feature Subset Selection (CFS) [49]; (ii) A subset of features is selected using Chi-square method that evaluates features’ chi-squared statistic with respect to the class label [50]; (iii) The conditional entropy of class given the selected features is minimized using the Gain ratio method [51]; (iv) The importance of a test feature set is evaluated using the RELIEF method that examines the difference of Euclidean distances for randomly selected samples with the nearest samples of the same and different classes using the test feature set [52], (v) The SVM-based ranker, in which the features are ranked by the square of their weights assigned by the SVM classifier [53]. For completeness of comparisons, we also employed popular sparsity imposing regression methods including LASSO [54] and logistic regression and selected the top-20 features with highest absolute coefficients in the model. We also included the results for the classification accuracy using all 360 features that are shown by NoFS in Figure 1 and Figure 2. Finally, the results are also compared with our recently developed Shapley-based coalition game theoretic feature selection method [27,29].

In order to compare the performance of the aforementioned feature selection methods, Bayes-Net with 10-fold cross validation is selected as a representative classifier to classify the alarms into false and true alarms. It is worth mentioning that definition of the proposed feature selection method is independent of the choice of classifier technique and it can be applied to all classification techniques. The classification success rates, and the sensitivity and specificity for all aforementioned feature selection methods are presented in Figure 1 and Figure 2.

The results in Figure 1 represent the alarm classification success rate which is the ratio of successfully classified alarms. Figure 1, represents the specificity and sensitivity of the classifier using features reported by different methods. Sensitivity is calculated as the ratio of recognized true alarms to the number of all true alarms. Likewise, specificity is calculated as the ratio of recognized false alarms to the number of all false alarms. In other words, a higher sensitivity is desired for not missing a true alarm and an acceptable level of specificity is required not to report a false alarm. The trained classifier shows a better sensitivity compared to the majority of feature selection methods, which is desired since missing a true alarm may have catastrophic consequences. It is worth mentioning that the obtained results cannot be directly compared to the top entries from the Physionet Challenge 2015, since in this paper, we have a total of (220) samples for both training and test purposes, which is substantially less than those reported works which had access to 750 training samples and 500 test samples.

The results show that the proposed algorithm when combined with Bayes Net classifier outperforms the majority of other feature selection methods in recognizing true and false alarms with a low computation complexity. Interestingly, the false alarm recognition rate (specificity) is substantially improved compared to the best competitors methods, while the true alarm recognition (sensitivity) remains almost at the same level. The low success rate for NoFS is somewhat expected and demonstrates the value of feature selection, since incorporating all features in classification not only increases the time and computational load of the classifier, but also decreases the classification accuracy due to the well-known over-fitting problem. It is also observed that the proposed method provides a similar level of accuracy compared to our previously developed coalition-based game theoretical feature selection method using Shapley value. However, the Banzhaf-based coalition game includes much less computational power. In Shapley-based coalition game, the marginal importance of a feature $F_{i}$ when joining a coalition S with |S| members is calculated by checking all $2^{\left|S\right|}$ permutations. However, in Banzhaf-based coalition game, in order to evaluate marginal importance of a feature $F_{i}$ with respect to coalition S, we examine interdependency of this feature with |S| members, that requires much less calculations. In summary, the key advantages of the proposed feature selection method are: (i) providing a comparable results to the best feature selection methods including CFS, (ii) considering the linear and non-linear correlation among the extracted features extracted from different signals beyond the commonly used pairwise correlations (which are in this application), and (iii) offering a relatively low computational complexity compared to previously proposed coalition game-theoretic approaches (such as Shapley-based method proposed.

**Figure 3:**
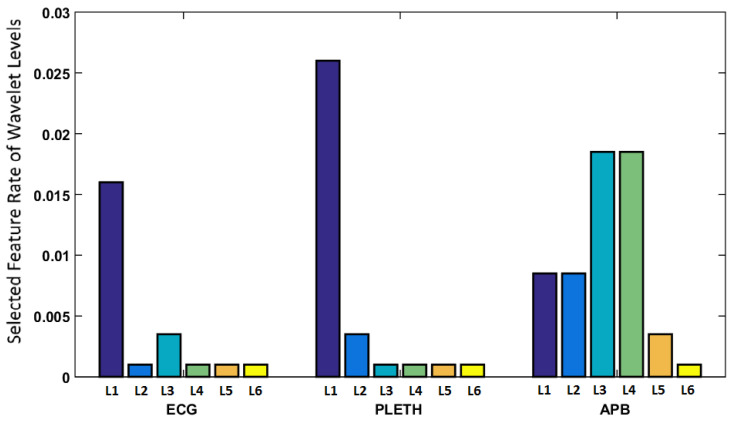
Relative appearance of selected features in different levels of wavelets for the vital signals.

Figure 3 demonstrates the rate of selected features from each of the wavelet levels for all signals. As can be seen, the features in low levels of ECG, corresponding to smoother waves such as *P* and *T* have proved to be more significant for this decision making task. A similar observation can be made regarding the low level features of PLETH. However, the medium levels of ABP appear to be selected more frequently in the process. The frequencies of variations in these levels of wavelet decomposition seem to correspond to the informative patterns in dicrotic notch.

**Figure 4:**
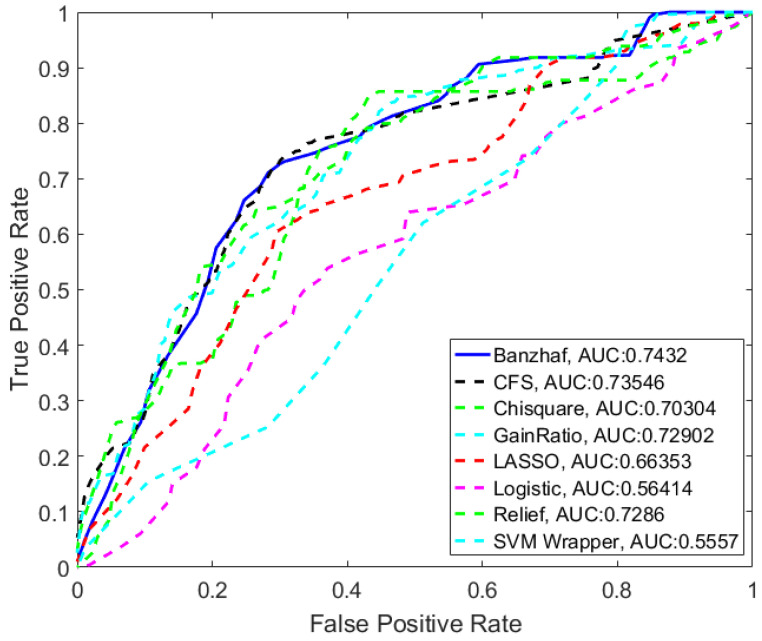
ROC curve for different feature selection methods.

Figure 4 compares the ROC (receiver operating characteristic) curve for different feature selection methods using Bayes-Net classification with 10-fold cross validation. As can be seen in Table 3, the proposed method that is based on calculating Banzhaf power achieves the highest area under the ROC curve (AUC = 0.7432) compared to well-known feature selection techniques.

Finally, we note that the above results are provided for 220 samples with valid ECG II, PLETH, and ABP signals, combining all alarm types, since the sample size for specific alarms are too small. Out of 220 patients with three recorded signals, the number of available samples for Asystole, Extreme Bradycardia, Extreme Tachycardia, Ventricular-Flutter/Fibrillation, and Ventricular-Tachycardia is 34 (4 true and 30 false alarms), 30 (21 false and 9 true alarms), 15 (14 false and 1 true alarms), 17 (12 false and 5 true alarms)and 124 (106 false and 18 true alarms), respectively. However, for the sake of completeness, we present the results of our proposed method for the fifth alarm: Ventricular-Tachycardia, which includes 124 samples. We used the top-20 features reported by the proposed method. Table 4 presents the obtained accuracy, precision and recall rate using different classification methods. The obtained accuracy 85.5% rate is higher than that of the entire dataset (77.6%), due to the intrinsic differences among the signals corresponding to different alarm types. Therefore, for larger datasets, performing per-alarm analysis is desired. It is interesting to see that most classification algorithms perform almost equally. In addition, BayesNet significantly outperforms Naive Bayes where the relations among features are not considered.

### Limitations

The proposed feature selection method can be utilized along with different classification methods. Here, we note that it is a known fact that different feature selection methods may perform differently when applied to different classifiers. To investigate this problem, we have tried the performance of our feature selection method over different classifiers including Random Forest, Naive Bayes, Sequential Minimal Optimization (SMO), and J48 decision tree. We observe that our proposed method performs almost equivalently to other feature selection methods, and the improvement is considerable for the Bayes-Net classifier. The justification for this observation is that Bayes-Net considers the relations among the features and hence using Banzhaf power which selects features with stronger synergistic powers yields better results. Therefore, to benefit from this proposed method, it is more advantageous to use it with classifiers that consider inter-feature dependencies. It is worth mentioning that the numerical results are reported for a small and relatively unbalanced dataset of 220 patients with three signals of ECG II, ABP and PLET. Out of these samples, 50 are false and 170 are true alarms.

## 6. Conclusions

One of the critical concerns in intensive care units that has not been resolved yet is the high false alarm rate. In this paper, we proposed a novel coalition game theoretic-based feature selection method to detect the false alarms. The proposed method accounts for information-theoretic correlation among the features in all possible coalitions of them. This feature selection problem is defined as a *simple* coalition game, where the average contribution of each feature (game player) is determined by Banzhaf power. A feature is defined to play a critical role in a coalition if it increased the relevancy of the coalition on target class and also was interdependent on more than half of the coalition’s members. The numerical results presented in this paper, calculated using Bayes-Net classifier, showed the superiority of the proposed method over existing feature selection methods such as Gain Ratio, Chi-square, and Relief methods in terms of false alarm detection as well as area under the ROC curve. It should be noted that the proposed method can be applicable to commonly used classifiers that require feature selection. However, it is more likely that the proposed method outperforms other feature selection techniques when integrated with classifiers which consider the inter-feature dependencies.

## Figures and Tables

**Table 1:** Alarms definition [32].

Alarm Type	Definition
Asystole	No heartbeats for at least 4 s
Extreme Bradycardia	Heart rate less than 40 bpm for 5 consecutive beats
Extreme Tachycardia	Heart rate higher than 140 bpm for 17 consecutive beats
Ventricular Tachycardia	At least 5 ventricular beats with heart rate higher than 100 bpm
Ventricular Flutter/Fibrillation	Fibrillatory, flutter, or oscillatory waveform for at least 4 s

**Table 2:** Information-theoretic and statistical features of wavelet coefficients.

No.	Feature	No.	Feature	No.	Feature
1	mean	8	std (σ)	15	Interquartile
2	mode	9	$\mu_{3}$		Range
3	median	10	$\mu_{4}$	16	Shannon Ent.
4	max	11	coef. of var	17	Log Eng.
5	min	12	kurtosis	18	$n_{T}\left(max\left\{X_{i}\right\}/2\right)$
6	range	13	skewness	19	$n_{T}\left(\sqrt{\sum X_{i}^{2}}\right)$
7	variance	14	H mean	20	$n_{T}\left(5\sqrt{\sum X_{i}^{2}}\right)$

Coef. of var: coefficient of variation; Shannon Ent.: Shannon entropy ; Log Eng.: Log. Energy.

**Table 3:** Comparison of area under ROC curve for different feature selection methods.

Feature Selection Method	Area under ROC Curve (AUC)
Proposed method based on Banzhaf power	**0.7432**
CFS	0.73546
ChiSquare	0.70304
GainRatio	0.7290
LASSO	0.66353
Logistic	0.56414
Relief	0.7286
Wrapper	0.5557

**Table 4:** The performance of the proposed method in terms of classification accuracy, precision and recall rate for samples with alarm type of Ventricular-Tachycardia arrhythmia using different classifiers. The dataset includes 124 samples with 106 false and 18 true alarms.

Classification Method	Accuracy	Precision	Recall
Bayes Net	85.48	0.73	0.86
Rotation Forest	85.48	0.73	0.86
Naive Bayes	72.6	0.74	0.73
IBK	85.48	0.73	0.86
J48 (tree)	85.48	0.73	0.86

## Abbreviations

The following abbreviations are used in this manuscript:

ABP	Aeterial Blood Pressure
CFS	Correlation-based Feature Subset Selection
DWT	Discrete Wavelet Transform
ECG	Electrocardiogram
FFT	Electrocardiogram
FIR	Finite Impulse Response
ICU	Intensive Care Unit
LDA	Linear Discriminant Analysis
MIMIC	Multiparameter Intelligent Monitoring in Intensive Care
MLP	Multilayer Perceptron
PPG	photoplethysmogram
PCA	Principle Component Analysis
PPI	Protein-Protein Interaction
SQI	Signal Quality Indecies
SVM	Support Vector Machine
TU	Transferable Utility
HRV	Heart Rate Variability
